# Characterizing heterogeneity in motivational impairments in psychosis

**DOI:** 10.3389/fpsyt.2025.1485460

**Published:** 2025-05-21

**Authors:** Kathryn E. Lewandowski, Jintian Luo, Beier Yao, Alexis E. Whitton

**Affiliations:** ^1^ Schizophrenia and Bipolar Disorder Program, McLean Hospital, Belmont, MA, United States; ^2^ Department of Psychiatry, Harvard Medical School, Boston, MA, United States; ^3^ Black Dog Institute, University of New South Wales, Sydney, NSW, Australia

**Keywords:** reward, motivation, schizophrenia, bipolar disorder, psychosis, cluster analysis

## Abstract

**Introduction:**

Motivational impairments are a hallmark symptom of psychotic disorders. However, motivation is a multidimensional construct believed to be underpinned by different neural mechanisms and differentially impaired both between and within diagnostic groups. We used a data driven approach to identify different motivational profiles in people with psychosis.

**Methods:**

Participants (n=242) included people with a diagnosis of a DSM-V schizophrenia spectrum disorder (SSD; n=95), mood disorder with psychosis (MDP; n=95), and healthy controls (n=52). Participants were assessed using the Behavioral Inhibition/Behavioral Activation Scales (BIS/BAS), measures of clinical symptoms, assessments of hedonic capacity (anticipatory and consummatory pleasure; TEPS), and a behavioral task of effort expenditure for reward. The four BIS/BAS subscales from the patient groups were normed to the controls and entered first into a hierarchical cluster analysis, and then into K-means cluster analysis for the final cluster solution.

**Results:**

A four-cluster solution best fit data, reflecting: a High Avoidance group (n=56); a High Approach group (n=66); a Low Approach/High Avoidance group (n=26); and a Low Approach group (n=35). Diagnostic groups were represented in each cluster. Clusters differed on depression and anxiety severity on both interview-based and self-report measures, as well as on anticipatory and consummatory pleasure. Contrary to our hypothesis, groups did not differ on a measure of community functioning.

**Discussion:**

These findings suggest that aspects of approach and avoidance motivation may be both uniquely and additively associated with anxiety, depression, and hedonic experiences. Characterization of motivational profiles may help parse heterogeneity in motivation and predict other important aspects of illness.

## Introduction

Motivational deficits are a hallmark of schizophrenia and related disorders and are associated with poor community outcomes and reduced treatment adherence and efficacy (1–8). However, considerable heterogeneity in motivational impairments exists across the psychosis spectrum both between and within diagnoses. Data driven approaches like cluster analysis have been used to identify distinct subgroups of patients sharing similar symptom profiles in other multidimensional constructs such as cognition, and have revealed clinical and neurobiological differences amongst clusters (9–12). Such an approach may also hold utility in parsing the heterogeneity in motivational impairments among those with psychosis spectrum disorders, which may similarly be associated with relevant clinical and biological features and have implications for targeted interventions.

Motivation is a multidimensional construct comprised of measurable and separable domains including approach and avoidance motivation, which are believed to represent distinct trait-like constructs underpinned by separable neurobiological mechanisms (13, 14). Approach motivation is associated with reward sensitivity and other appetitive processes, whereas avoidance motivation is associated with inhibition of behavior driven by aversion to threat or punishment (15). At the group level, findings show mixed patterns of impaired and preserved aspects of motivation in people with psychosis (5, 16–22). Studies finding group differences between patients with schizophrenia (SZ) and control groups typically report heightened avoidance sensitivity and/or diminished approach motivation (21–23) using the Behavioral Inhibition Scale and Behavioral Activation Scales (BIS/BAS); however, other studies report no differences from controls (20, 21). Similarly, studies of group differences in people with mood disorders such as bipolar disorder (BD) report elevated avoidance motivation as compared to controls (18, 19), although findings are also mixed (16). In contrast with SZ, approach motivation has been found to be elevated in BD in some reports (16–19), and elevated BAS subscales predict shorter time to BD Type I diagnosis in people with bipolar spectrum disorders (16). The one study we are aware of directly comparing BAS in people with SZ and BD found reduced BAS reward responsiveness in SZ compared to BD; in this report people with BD did not differ from controls (22).

The association between BIS/BAS scores and other aspects of reward and motivation as well as state clinical symptoms or cognitive functioning is unclear. For instance, higher BIS scores have been associated with lower negative symptoms and higher anxiety in SZ, but not with positive symptoms or depressed mood (20, 21). Higher BAS was associated with higher anticipatory and consummatory pleasure in SZ (20, 22, 24), but was not related to anxiety, depressed mood, or positive symptoms (20, 21). In contrast, in people with BD BIS/BAS scores have been associated with mania, depression, and anxiety in several reports (16–19, 25), but not with anticipatory or consummatory pleasure (22). In terms of functional outcomes, elevated BIS scores have been associated with poor outcomes in people with SZ (26). Because individuals may have differing profiles of approach and avoidance motivation, being able to examine subgroups sharing similar patterns across the BIS/BAS scales may clarify associations of unique combinations of these traits with clinical, functional, and biological features.

Data-driven approaches to characterizing heterogeneity in approach and avoidance motivation may reveal distinct subgroups, potentially explaining some of the inconsistencies reported at the group-level. Only one study to date has examined subgroups of people with SZ with similar BIS/BAS motivational profiles using cluster analysis. Felice Reddy and colleagues (27) identified a five-cluster solution: High BIS/Moderate BAS, Moderate BIS/High BAS, Moderate BIS/Low BAS, Low BIS/Moderate BAS, and Low BIS/Low BAS. Groups differed in symptom severity; both High BIS/Moderate BAS and Low BIS/Low BAS subgroups exhibited significantly higher negative symptoms – the former had elevated social avoidance motivation while the latter had a lack of social approach motivation. On the other hand, Moderate BIS/High BAS and Low BIS/Moderate BAS subgroups endorsed better functioning with low negative symptom scores, with the former also endorsing high anticipatory pleasure. These findings illustrate the importance of identifying distinct BIS/BAS profiles within people with psychosis, which may be associated with specific symptoms and functioning through different motivational pathways.

No studies to date have examined motivational profiles using cluster analysis in a transdiagnostic psychosis sample of people with psychosis, despite substantial clinical and biological overlap across the psychotic disorders. In the current study, we used data-driven cluster analysis techniques in a large, well characterized sample of patients across the psychosis spectrum to identify distinct motivational profiles using the BIS/BAS scales as described in Felice Reddy et al. (27). We then compared emergent clusters on demographic, clinical, and functional measures, and other aspects of motivation and reward. We hypothesized that distinct motivational profiles would emerge transdiagnostically, and that profiles would differ on clinical measures and other measures of reward and motivation including hedonic capacity and effort expenditure for reward.

## Method

### Participants

Participants (n=242) included people with a diagnosis of a DSM-V schizophrenia spectrum disorder (SSD) including schizophrenia, schizoaffective disorder, schizophreniform disorder, other specified psychotic disorder (n=95), DSM-5 mood disorders with psychosis (MDP) including bipolar I disorder with psychosis and major depressive disorder with psychotic features (n=95), and healthy controls (n=52). Eligible participants were adults ages 18-60; all participants were stable outpatients at the time of testing, defined as no inpatient admission or medication changes within the past month, and did not endorse symptoms consistent with a current mood episode during screening procedures. Exclusion criteria included history of head injury with loss of consciousness, history of seizure disorder, lifetime history of DSM-5 severe substance use disorder, and any DSM-5 substance use disorder within the past month. Diagnosis was ascertained using the SCID-5 administered by trained research staff in the context of one of several ongoing research studies in our group. In a small number of cases (*n=*9), a SCID was unavailable and diagnosis was determined using a combination of participant self-report and available medical records. Healthy controls had no lifetime history of any DSM-5 psychiatric diagnosis or psychiatric treatment. All participants provided written informed consent. All procedures were approved by the McLean Hospital Institutional Review Board.

### Materials

#### Motivation and reward measures

Approach and avoidance motivation were assessed using the Behavioral Inhibition Scale/Behavioral Activation Scale (BIS/BAS) (28). The BIS/BAS is a self-report measure that includes items assessing approach motivation across three subscales including Drive, Fun Seeking, and Reward Responsiveness, and avoidance sensitivity in a single subscale that measures behavioral and affective inhibitory responses. Hedonic experience was assessed using the Temporal Experience of Pleasure Scale (TEPS) (29). The TEPS includes 10 items assessing anticipatory pleasure and 8 items assessing consummatory pleasure. The Effort Expenditure for Reward Task (EEfRT; (30)) was administered to assess motivated behavior during a standardized computer-administered task. On each trial participants must choose to engage in an easy action (use dominant index finger to press a key 30 times in 10 seconds) or a difficult action (using non-dominant little finger to press a key 100 times in 21 seconds). Prior to choosing, participants are shown the probability (12%, 50%, 88%) of receiving an imaginary monetary reward, and the amount of money they could win ($1 for easy; $1.12-$4.12 for hard). Participants complete as many trials as possible in 20 minutes. Based on our previous work we extracted two EEfRT variables reflecting the proportion of hard choices made as a function of probability level (EEfRT Prob) and reward value (EEfRT Val) (31).

#### Clinical and functional measures

Clinical and functional assessment included interview-based measures of depression using the Montgomery-Asberg Depression Rating Scale (MADRS) (32), symptoms of mania using the Young Mania Rating Scale (YMRS) (33), symptoms of psychosis using the Positive and Negative Syndrome Scale (PANSS) (34). Anxiety was assessed using the self-report State/Trait Anxiety Inventory (STAI). Community functioning was evaluated using the Multnomah Community Ability Scale (MCAS) (35), an interview-based assessment of several aspects of community functioning including social interest and engagement, independence in daily living, and instrumental role functioning. We used an abbreviated version excluding items related to symptom severity (36). Items are scored 1-5; higher scores indicate better functioning.

#### Procedures

Assessments occurred over one or two sessions based on participant preference in the context of a larger study of reward and motivation in transdiagnostic psychotic disorders. Self-report questionnaire measures and the EEfRT task were completed via computer; interview measures were completed by trained research staff.

#### Statistical approach

Participant groups were compared on demographic and clinical measures using ANOVA, χ2, or t-tests as appropriate. Cluster analyses were performed in Stata 16.1 (StataCorp LP, USA). The four BIS/BAS subscale (Drive, Fun Seeking, Reward Responsiveness, and Inhibition) were standardized to the HC means, and z scores for each domain were entered into the cluster analysis to ensure that each contributed equally to the distance measure. A two-step approach was used; first, clusters were derived using Ward’s linkage with squared Euclidean distance, an agglomerative hierarchical clustering technique. Data were then entered into a K-means cluster analysis, and model fit was tested using discriminant function analysis and elbow test. Emergent clusters were compared on demographic, clinical, and motivation and reward variables using analysis of variance (ANOVA) or χ2. *Post-hoc* t tests with Bonferroni correction were conducted to examine pairwise relationships between clusters. Lastly, we conducted a series of linear regressions predicting symptom levels by diagnosis, cluster, and sex.

## Results

### Participant characteristics

Group comparisons by diagnosis showed that groups differed on age, sex, race, and years of education (**Table 1**). In terms of clinical measures, patient groups differed from each other on severity of positive and negative symptoms of psychosis (SSD>MDP) and depression (MDP>SSD) but not on mania or state or trait anxiety (**Table 1**).

**Table 1:** Demographic, clinical and motivation measures by diagnosis.

	SSD (n=95)	MDP (n=95)	HC (n=52)	Test	*Post-hoc*
**Age**	36.0 (11.5)	32.3 (10.3)	37.6 (14.3)	F=4.21*	**MDP<HC**
**Race** **(% Caucasian)**	61%	78%	68%	Chi2 = 6.65*	**SZ<MDP**
**Sex (% Female)**	35%	54%	43%	Chi2 = 6.88*	**SZ<MDP**
**Education (years)**	14.2 (2.7)	15.8 (2.2)	16.1 (2.2)	F=14.80***	**SZ<HC, MDP**
**MADRS**	14.2 (9.0)	17.6 (11.4)	–	t=2.26*	**SSD<MDP**
**YMRS**	11.5 (6.8)	10.7 (9.3)	–	t=-0.68	
**PANSS Positive**	15.3 (5.4)	12.4 (4.7)	–	t=-3.83**	**MDP<SSD**
**PANSS Negative**	14.8 (5.3)	11.3 (3.8)	–	t=-5.31***	**MDP<SSD**
**PANSS General**	32.0 (8.3)	29.5 (6.9)	–	t=5.17*	**MDP<SSD**
**STAI Trait**	45.0 (13.6)	49.1 (13.7)	31.4 (7.4)	F=33.79***	**HC<SSD, MDP**
**STAI State**	39.3 (12.4)	41.0 (12.4)	29.1 (7.1)	F=19. 35***	**HC<SSD, MDP**
**CPZE**	357.4 (350.6)	183.5 (271.4)	–	t=-3.75**	
**MCAS**	46.3 (5.3)	49.7 (4.0)	–	t=4.99***	**SSD<MDP**
**BAS Drive**	11.67 (2.51)	10.94 (3.00)	11.88 (2.25)	F=2.61	
**BAS Fun**	11.18 (2.65)	11.62 (2.83)	11.68 (2.47)	F=0.83	
**BAS Reward**	17.01 (2.25)	16.57 (2.66)	17.72 (2.02)	F=3.80*	**MD<SSD, HC**
**BIS**	20.81 (4.08)	22.18 (4.72)	18.98 (4.11)	F=8.77***	**MDP>SZ>HC**
**TEPS Ant**	41.5 (8.5)	40.6 (10.3)	46.5 (6.9)	F=7.80***	**SSD, MDP<HC**
**TEPS Con**	35.9 (6.8)	36.7 (7.5)	39.2 (6.2)	F=3.72*	**SSD<HC**
**EEfRT Prob**	0.11 (0.24)	0.30 (0.29)	0.34 (0.26)	F=17.67***	**SSD<MDP, HC**
**EEfRT Value**	0.15 (0.23)	0.26 (0.21)	0.29 (0.22)	F=9.43***	**SSD<MDP, HC**

*p<.05, **p<.01, ***p<.001. The bolded items indicate significant results.

In terms of reward and motivation measures, groups did not differ on BAS Drive or Fun Seeking. The MDP group scored lower on BAS Reward Responsiveness than the SSD or HC groups, and highest on BIS, followed by the SSD and then the HC group. Both patient groups scored lower than the HC group on Anticipatory Pleasure. The SSD group scored lower than the HC group on Consummatory Pleasure; SSD and MDP groups did not differ. The SSD group scored lower than the MDP and HC groups on EEfRT Probability and Value measures; the MDP and HC groups did not differ (**Table 1**).

### Cluster solution

**Figure 1:**
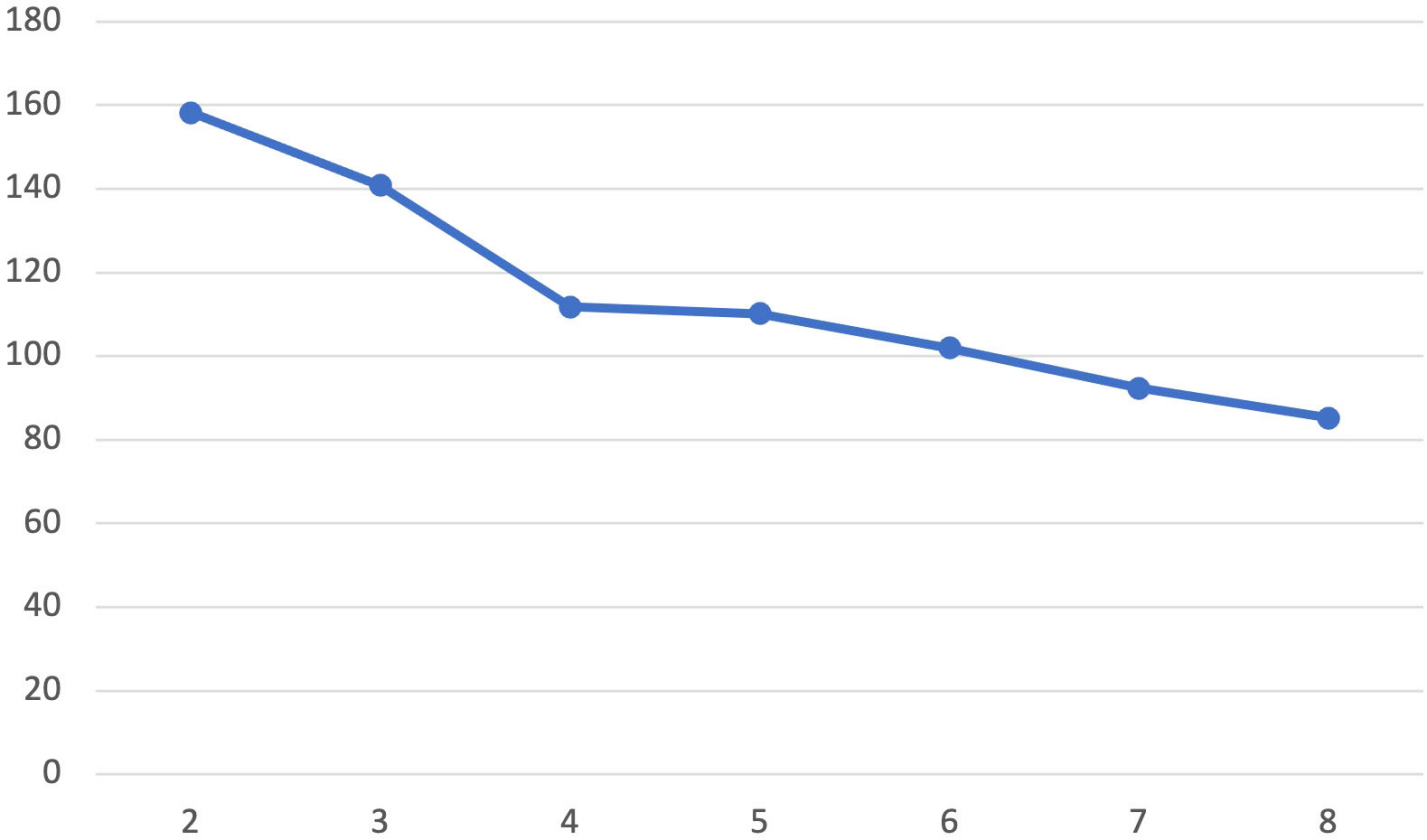
Elbow test by cluster solution. Plot of within-cluster sum of squares for different values of k derived from K-means cluster analyses.

The resulting dendrogram from the Ward’s method cluster analysis showed evidence supporting a 2, 3 or 4 clusters solution. K-means clustering was undertaken examining model fit of 2-8 clusters. An “elbow test,” plotting the within cluster sums of squares for each cluster solution (**Figure 1**) showed an “elbow” at four clusters. Canonical linear discriminant analysis found excellent differentiation at each level in the four-cluster solution (F=53.21, p<.0001 – F= 7.38, p=.0001), and assuming equal prior probabilities clusters accurately classified 96.3% (Cluster 3) to 100% (Cluster 1, 2, and 4) of participants.

**Figure 2:**
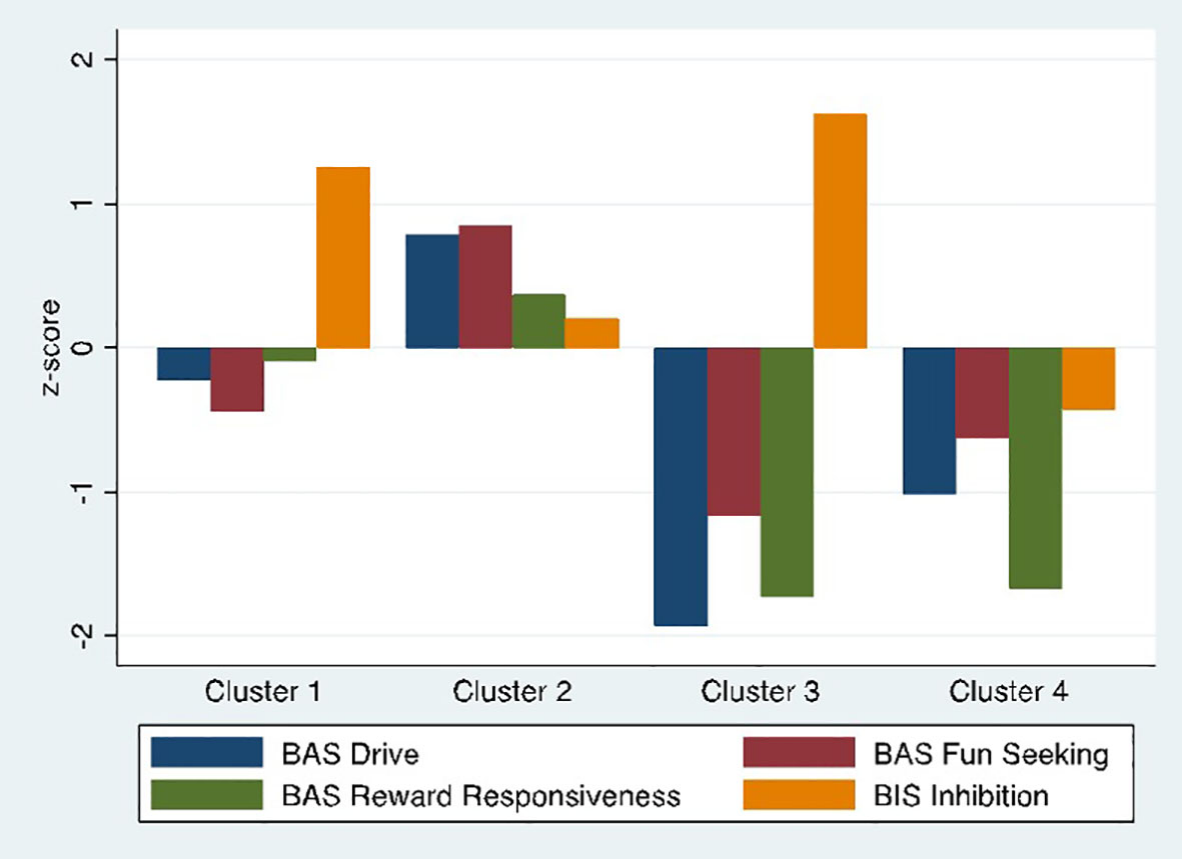
Final four cluster solution. BIS/BAS subscale scores by cluster. Scores are standardized to the healthy control sample.

BIS/BAS measures by cluster showed four distinct profiles (**Figure 2**): a High Avoidance group (n=56) with BIS scores more than 1 standard deviation above HC mean and no difference from HCs on BAS scales (Cluster 1); a High Approach group (n=66) with nearly one standard deviation elevation on BAS Drive and Fun-Seeking and no difference from controls on BIS (Cluster 2); a Low Approach/High Avoidance group (n=26) with low scores on all BAS approach measures, particularly BAS Drive and Reward Responsiveness, and elevated BIS scores (Cluster 3); and a Low Approach group (n=35) with low BAS scores, particularly in BAS Drive and Reward Responsiveness but no difference from HCs on BIS (Cluster 4).

### Demographic and clinical variables by cluster

Clusters did not differ on age, race, or years of education; however, Clusters 1 (High Avoidance) and 3 (Low Approach/High Avoidance) contained a significantly greater proportion of females than did clusters 2 (High Approach) and 4 (Low Approach) (**Table 2**). In terms of clinical characteristics, clusters did not differ on positive or negative symptoms of psychosis, or symptoms of mania. Depression and PANSS General symptoms were most severe in Cluster 3 (Low Approach/High Avoidance), followed by Cluster 1 (High Avoidance). Both state and trait anxiety were highest in the two clusters exhibiting high avoidance (Clusters 1 and 3). Community functioning did not differ by cluster.

**Table 2:** Demographic and clinical measures by cluster.

	Cluster 1 (n=56)	Cluster 2 (n=66)	Cluster 3 (n=26)	Cluster 4 (n=35)	Test	*Post-hoc*
**Age**	34.2 (10.8)	32.6 (9.2)	38.2 (13.2)	34.1 (11.9)	F=1.62	
**Race (% Caucasian)**	71%	62%	80%	66%	Chi2 = 3.10	
**Sex (% Female)**	57%	33%	73%	26%	Chi2 = 20.52***	**2,4<1,3**
**Education (years)**	15.1 (2.6)	14.9 (2.6)	15.5 (2.8)	14.8 (2.2)	F=0.39	
**MADRS**	17.6 (9.9)	12.8 (8.8)	24.8 (10.7)	12.3 (9.1)	F=12.18****	**3>1>2,4**
**YMRS**	11.0 (7.3)	11.9 (8.7)	10.0 (7.6)	11.0 (8.9)	F=0.37	
**PANSS P**	13.8 (5.0)	14.0 (5.5)	13.2 (4.7)	14.7 (5.8)	F=0.46	
**PANSS N**	13.2 (5.1)	12.4 (4.9)	12.5 (3.9)	14.3 (5.4)	F=1.22	
**PANSS G**	32.4 (8.2)	29.4 (7.2)	34.2 (7.5)	28.2 (7.0)	F=4.79**	**3>1,2,4**
**STAI State**	43.8 (11.5)	36.4 (11.8)	45.1 (13.4)	36.8 (10.4)	F=6.53****	**1,3>2,4**
**STAI Trait**	52.5 (12.2)	40.0 (11.4)	58.1 (14.0)	42.7 (11.3)	F=20.22****	**1,3>2,4**
**CPZE**	328.5 (340.8)	231.8 (288.4)	187.0 (188.2)	320.0 (358.5)	F=1.86	
**BACS**	41.6 (12.0)	38.0 (14.0)	47.3 (11.2)	43.9 (15.0)	F=3.57*	**2<3**
**MCAS**	47.7 (5.0)	48.7 (5.1)	48.0 (4.3)	47.4 (5.3)	F=0.67	

* p<.05, ** p<.01, *** p<.001, **** p<.0001. The bolded items indicate significant results.

**Figure 3:**
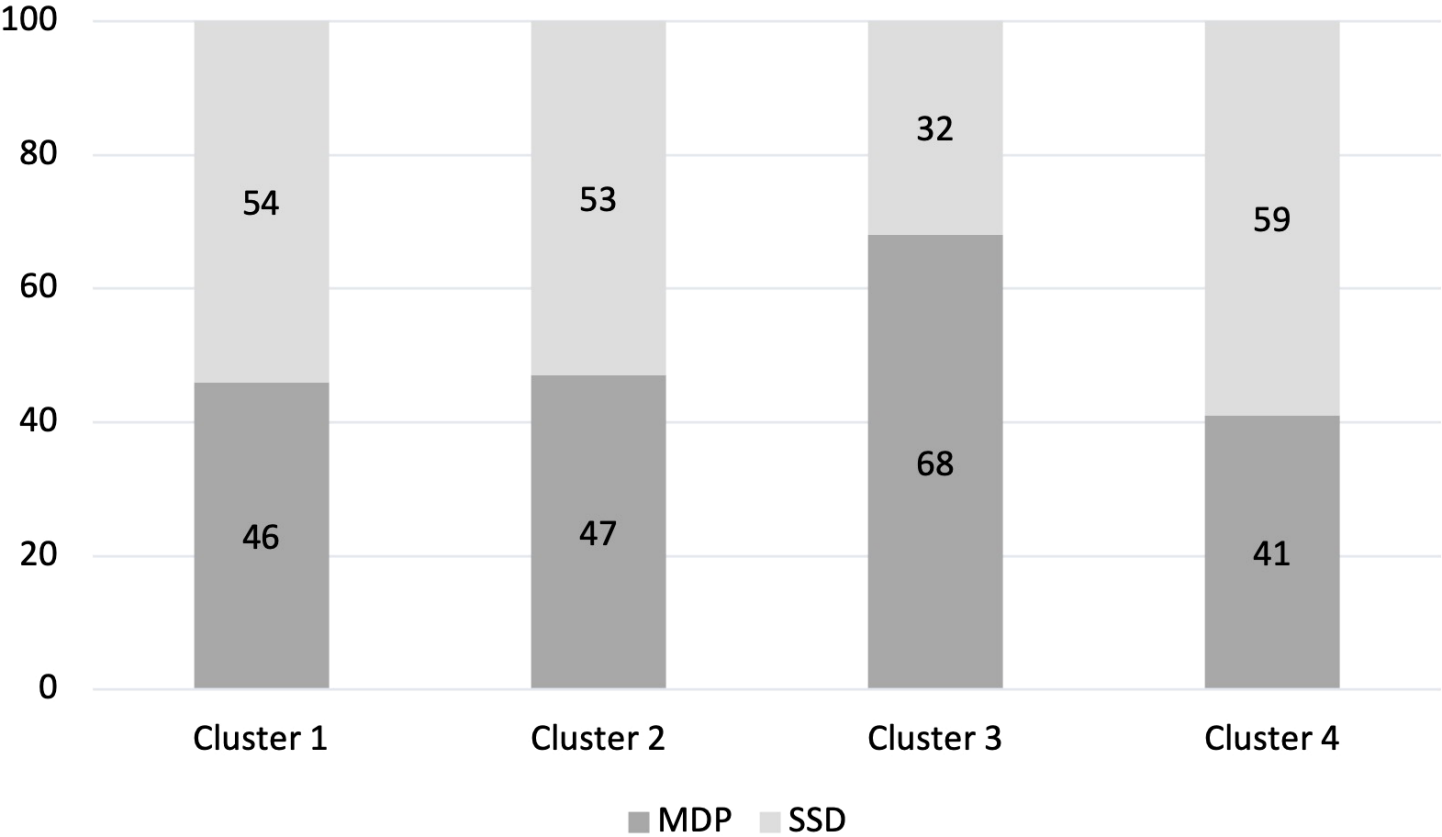
Proportion of cluster classified as MDP or SSD (%). Percentage of each Cluster made up of people with an MDP diagnosis and people with an SSD diagnosis.

Both SSD and MDP participants were assigned to each cluster (χ2 = 5.46, *p*=.14). Participants with MDP were slightly but non-significantly overrepresented in Cluster 3, whereas participants with SSD were slightly but non-significantly overrepresented in Cluster 4 (**Figure 3**).

### Motivation, reward measures

Anticipatory and consummatory pleasure were highest in Cluster 2 (High Approach). Clusters 3 and 4, both characterized as having low approach motivation, were significantly lower on Consummatory pleasure than Cluster 2. Cluster 3 showed the lowest Anticipatory pleasure, followed by Clusters 1 and 4. EEfRT measures did not differ by cluster.

In an exploratory analysis, we examined associations between BIS/BAS scales, negative symptoms, and EEfRT within clusters to examine the possibility that the null omnibus tests of group difference obscured within-group associations. In Cluster 1, negative symptoms were correlated with BIS (r=-.29, p=.03). Negative symptoms were not correlated with any of the BIS/BAS scales in the other three clusters. Similarly, EEfRT scores were not correlated with any of the BIS/BAS scales in any cluster. Overall, these findings suggest that associations between BIS/BAS, negative symptoms, and EEfRT task performance are largely consistent across the clusters.

### Linear regression analyses

Given the sex differences by cluster, we conducted t-test to determine across the patient sample whether depression and anxiety symptoms differed by sex. We repeated these analyses replacing anxiety and depression measures with TEPS anticipatory and consummatory pleasure. We found significant sex differences in STAI State (t=2.95, p<.01), STAI Trait (t=4.16, p<.0001), and MADRS (t=6.17, p<.0001), with female participants reporting higher symptom levels in all cases. Anticipatory pleasure also differed by sex (t= -2.29; p=.02) and Consummatory pleasure differed at the trend level (t=-1.73; p=.08). Thus, we conducted a series of linear regressions to examine effects of sex, diagnosis, and cluster on anxiety and depression symptoms, to determine whether cluster assignment remained a significant predictor after accounting for the effects of sex and diagnosis. All three overall models were significant (p<.001-p<.0001), and cluster assignment was a significant predictor of MADRS (t=-2.65, p<.01), STAI State (-1.92, p<.05), and STAI Trait (t= -3.52, p<.001) after accounting for the effects of sex and diagnosis. Similarly, both TEPS models were significant (p<.0001), and cluster assignment was a significant predictor of both TEPS Anticipatory (t= 9.45, p<.0001) and Consummatory pleasure (t= 4.48, p<.0001) after accounting for the effects of sex and diagnosis (**Table 3**).

**Table 3:** Motivation and reward measures by cluster.

	Cluster 1 (n=56)	Cluster 2 (n=66)	Cluster 3 (n=26)	Cluster 4 (n=35)	Test	*Post-hoc*
**TEPS Ant**	41.1 (7.7)	47.2 (6.4)	30.7 (7.7)	37.2 (9.4)	F=33.11****	**2>1,4>3**
**TEPS Con**	36.9 (6.2)	38.5 (6.9)	32.7 (7.3)	33.3 (7.3)	F=7.10***	**1,2>3,4**
**EEfRT Prob**	0.21 (0.28)	0.20 (0.29)	0.26 (0.29)	0.19 (0.29)	F=0.74	**–**
**EEfRT Val**	0.17 (0.18)	0.19 (0.22)	0.29 (0.21)	0.20 (0.27)	F=1.79	**–**

*** p<.001. ****p<.0001.

The bolded items indicate significant results.

## Discussion

Motivation abnormalities are central to the psychosis syndrome and associated with disability and poor quality of life. However, considerable variability exists in motivational impairments that may be concealed by reliance on group level analyses. This variability may reflect meaningful differences in both phenotypic expression and underlying neurobiology. Here we identified four distinct motivational subgroups using a data-driven clustering approach.

Emergent clusters showed different patterns of symptoms and hedonic profiles suggesting different contributions of approach and avoidance impairments. In terms of symptoms, both state and trait anxiety were elevated in Clusters 1 and 3, the two clusters with high avoidance motivation. Despite significant differences between these clusters on approach motivation, they did not differ from each other on anxiety suggesting that high avoidance is related to both state and trait anxiety independent of impairments in approach motivation. Cluster 3 showed the most severe symptoms of depression, followed by Cluster 1 and lastly Clusters 2 and 4, suggesting that avoidance may be more strongly associated with depressive symptoms, and that high avoidance together with low approach motivation may show an additive effect on depression severity. In terms of symptoms on the PANSS, Cluster 3 had significantly higher general symptom severity than the other three clusters, which did not differ from each other. The PANSS General subscale inquiries about general psychopathology including experiences related to mood and anxiety. High scores in Cluster 3 may have been driven in part by these ratings. PANSS Positive and Negative subscales did not differ by cluster. Regression analyses controlling for sex and diagnosis revealed that cluster assignment remained a significant predictor of clinical symptoms.

Clusters also differed on measures of anticipatory and consummatory pleasure. Clusters 3 and 4, both characterized by decreased approach motivation, showed the greatest reductions in anticipatory and consummatory pleasure. Cluster 2 did not differ from controls on either scale, and Cluster 1 showed impairments in Anticipatory pleasure in between Clusters 3 and 4 and controls. These findings suggest that low approach motivation is associated with reductions in both anticipatory and consummatory pleasure, whereas elevated avoidance but intact approach motivation is associated with reduced anticipatory but not consummatory pleasure. Regression analyses controlling for sex and diagnosis revealed that cluster assignment remained a significant predictor of both anticipatory and consummatory pleasure.

People with SSD and MDP were represented in all clusters, suggesting that cluster membership is not simply recapitulating diagnostic groups. This also suggests that patterns of impairments in motivation and their associations with clinical symptoms can be found trans-diagnostically.

Overall, these findings suggest that people with a combination of increased avoidance motivation and decreased approach motivation experience the most severe symptoms of depression and anxiety, and the lowest anticipatory and consummatory pleasure. *Increased* avoidance motivation appears more strongly linked to anxiety and depression symptoms, whereas *decreased* approach motivation appears more strongly linked to hedonic capacity, particularly consummatory pleasure.

Interestingly, clusters did not differ on PANSS negative symptoms. One reason may be that PANSS does not capture the most up to date conceptualization of negative symptoms. More recently developed instruments such as the clinical assessment interview for negative symptoms (CAINS) or the brief negative symptom scale (BNSS), which produce subscale scores reflecting current conceptualizations of negative symptom subdomains, may reveal more specific associations with motivational profiles.

Several limitations should be considered when interpreting the current findings. First, the diagnostic groups were not well matched on age, sex, and race. Though the focus of the current study was not on comparisons between conventional diagnostic groups, this could nevertheless introduce unintended variance into the data and reduce the power of statistical analyses. One attempt to address this was to include sex as a covariate in the regression analyses, and results remained significant after controlling for variance due to sex differences and diagnostic group. Second, most of the patients participating in this study were taking psychotropic medications. The effect of medications may have restricted the range of certain symptoms and masked underlying associations with motivational profiles. Future studies utilizing unmedicated or at-risk samples could further illuminate the associations between motivation and clinical symptoms. Additionally, we did not have information about duration of illness, and thus cannot assess whether clusters differed in terms of length of illness. While indirect, age did not differ by motivation cluster. Lastly, we did not have information about comorbidities. Given differences in mood and anxiety symptoms by cluster, future work should examine the relative prevalence of comorbidities among motivation subgroups.

The BIS/BAS is a brief, easy to administer self-report questionnaire that may be able to identify people on the psychosis spectrum experiencing or at risk for mood and anxiety symptoms and reduced hedonic capacity based on motivational profiles, regardless of their primary clinical diagnosis. Such findings have implications for clinical applications. Additionally, approach and avoidance motivation are believed to be underpinned by separable neural systems. These findings of different patterns of association among motivation, pleasure, mood, and anxiety suggest that subgroups of patients may experience abnormalities in different neural circuits, which may hasten our understanding of motivational impairments in psychosis and have implications for mechanism-specific interventions.

## Data Availability

The original contributions presented in the study are included in the article/supplementary material, further inquiries can be directed to the corresponding author/s.
